# A smartphone aptasensor for fipronil detection in honey samples

**DOI:** 10.1007/s00216-023-05026-6

**Published:** 2023-11-09

**Authors:** Rossella Svigelj, Noemi Dassi, Andrea Gorassini, Rosanna Toniolo

**Affiliations:** 1 Department of Agrifood, Environmental and Animal Sciences, University of Udine, Udine, Italy; 2 Department of Humanities and Cultural Heritage, University of Udine, Udine, Italy

**Keywords:** Biosensors, Aptamer, Smartphone, Honey, Food analysis, Aptasensor

## Abstract

In this study, an electrochemical smartphone-based aptasensor for the determination of fipronil was developed by modifying a screen-printed carbon electrode (SPCE). Fipronil is a broad-spectrum insecticide that has been widely used in various applications such as agriculture, veterinary, and household pest control. Recently, its use has raised concerns over the potential impact on the environment and human health. The absence of effective methods for this purpose poses a significant obstacle. To tackle this problem, we have developed a cutting-edge aptamer-based portable sensor capable of rapidly and conveniently detecting fipronil in situ. Considering that the detection of small molecules, such as fipronil, can be a challenging task, a competitive replacement assay was set up based on the aptamer’s preference for the free form of fipronil over the immobilized one on the electrode. The analytical performance provided by the sensor on standard solutions of a known fipronil content made it possible to estimate a limit of detection (LOD) equal to 1.07 μg kg^−1^ and a limit of quantification (LOQ) of 3.21 μg kg^−1^. Selectivity tests were conducted using atrazine as a possible interferent. The use and performance of the developed portable aptasensor was assessed on honey samples, which were simultaneously analyzed using an HPLC–MS method. This aptasensor could be an affordable and effective tool for accurately quantifying fipronil not only in honey samples but also in other food products.

**Graphical abstract:**

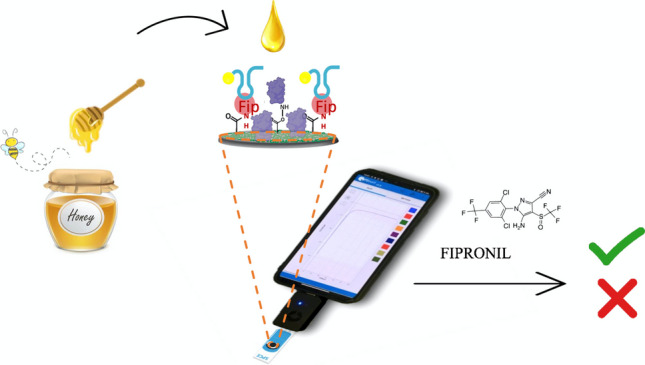

**Supplementary Information:**

The online version contains supplementary material available at 10.1007/s00216-023-05026-6.

## Introduction

The increased food demand, combined with the necessity of controlling insect-borne diseases, has stimulated the development of pesticides [1]. For many decades, the use of pesticides has been steadily increasing on agricultural land, especially in developing countries [2–4]. In Europe, the approval of the active substances is entrusted to the European Commission, which considers the scientific evaluation and peer reviews of the European Food Safety Authority (EFSA) [5]. In this context, legal limits and their control in marketed products are the main tools of risk management [6, 7]. European Commission promotes a road to the sustainable use of pesticides, an objective that is pursued within the Farm to Fork strategy, one of the central pillars of the Green Deal [8]. The concrete objective, to be achieved through the application of the Common Agricultural Policy, by 2030, consists of a 50% reduction in the use and risk of the most dangerous pesticides in the EU [9].

Hence, pesticides are among the analytes that attract the greatest attention in the field of food control [10, 11]. Among pesticides, fipronil garnered significant attention due to its involvement in the contamination of eggs in 2017 [12, 13]. Fipronil is a broad-spectrum insecticide that has been widely used in various applications such as agriculture, veterinary, and household pest control [14]. Recently, its widespread use has raised concerns over the potential impact on the environment and human health [15–18]. In the European Union, the maximum residue limit (MRL) for fipronil in food products is 5 μg kg^−1^ [19]; this limit applies to a wide range of food products, including eggs, meat, dairy, and certain fruits and vegetables [20].

Currently, the main analytical techniques exploited for the control of fipronil in different food matrices are gas chromatography and liquid chromatography, most of the time coupled to mass spectrometry [21–23]. However, traditional analytical methods require complex sample preparation and analysis procedures, specialized personnel, long times, and high costs and are not suitable as methods for in situ analysis [24]. Simpler alternative analytical tools could provide better monitoring of fipronil residues. Several techniques have been suggested as potential solutions, with notable examples such as Raman spectroscopy and enzyme-linked immunosorbent assays (ELISA), while these methods offer distinct advantages of analysis; they also exhibit certain limitations, some of which can be likened to those encountered in traditional methodologies [25, 26].

In this context, there is a significant opportunity for the application of biosensors, with aptasensors standing out as a noteworthy choice [27, 28]. Biosensors are incredibly versatile and powerful tools due to their low cost, ease of manufacture, rapid analysis, compact size, and exceptional detection capabilities [29–32]. However, the detection of small molecules, such as fipronil, can be a challenging task due to several factors [33, 34]. In fact, small molecules typically have low molecular weights and simpler structures compared to larger biomolecules such as proteins or nucleic acids. At the same time, when it comes to the most common detection strategies, sandwich assays cannot be employed due to the requirement of the target having at least two epitopes [35], which is an unrealistic condition in the case of small molecules [36]. On the other hand, a label-free assay, involving a signal directly correlated to the selective recognition of the molecule, runs the risk of exhibiting low sensitivity due to the small size of the target [37]. For these reasons, the use of simple and effective approaches, such as competitive assays, is of high interest [38, 39]. Among them, competitive replacement assays stand out as a compelling and effective method for detecting small molecules, as evidenced by their successful application in quantifying small compounds [40, 41].

Motivated by the potential benefits that a portable pesticide sensor could bring, here, we present a smartphone aptasensor that employs a competitive replacement assay approach to quantify fipronil. The primary impact of this research could be reflected in consumer safety, as well as in monitoring the potential environmental repercussions of pesticide residues in the ecosystem. To demonstrate the effectiveness of our work, we chose to test the aptasensor on honey samples.

## Materials and methods

### Reagents and materials

Two aptamers were used in the present work, one previously described in literature [42], and its corresponding form without primers, both biotinylated at the 5′ end and produced by Sigma-Aldrich (Milan, Italy). Table 1 shows the names and their respective sequences.

**Table 1:** Nucleotide sequence of aptamers

Name	Sequence
FPAP (fipronil aptamer with primers)	5′-[Btn]TGTACCGTCTGAGCGATTCGTACAGTTTCTGGAGGACTG GGCGGGGTGACGGTTATGAGCCAGTCAGTGTTAAGGAGTGC-3′
FPAS (fipronil aptamer Short)	5′-[Btn]AGTTTCTGGAGGACTGGGCGGGGTGACGGTTATG-3′

For the preparation of all the solutions and the cleaning operations, ultrapure water (*R* > 18 MΩ cm) was used, obtained with the Elga Purelab flex 4 system, Veolia Water Technologies (Italy). All reagents used were of analytical grade. Salts for buffer solutions, 2-aminobenzoic acid (2-ABA), 1-ethyl-3-[3-dimethylaminopropyl] carbodiimide hydrochloride (EDC), N-hydroxysuccinimide (NHS), fipronil, bovine serum albumin (BSA), tris(hydroxymethyl)aminomethane hydrochloride (Tris), and 3, 3′, 5, 5′-tetramethylbenzidine (TMB) were purchased by Sigma-Aldrich (Italy). Streptavidin conjugated horseradish peroxidase (HRP) was purchased by Merck (Italy). Ethanol was purchased from J.T. Backer (Italy), and methanol for HPLC–MS was manufactured by Honeywell, Riedel-de Haën (France). Atrazine was purchased by LGC Standards (UK). Screen-printed carbon electrodes (SPCEs) were purchased from DropSens (Metrohm, Italy).

Electrochemical measurements were performed by a portable potentiostat SensitSMART (Palmsens, Netherlands) managed via the PStouch app (version 2.8) and an Autolab PGSTAT204 potentiostat (Metrohm, Italy), managed by the Nova software (version 2.1). For the HPLC–MS analysis, it was necessary the purification of the samples through solid-phase extraction (SPE) cartridge C18, Isolute (1 g / 6 mL) of Biotage (Italy). The UHPLC instrument used was a Dionex Ultimate 3000 (Thermo Scientific, San Jose, CA, USA) equipped with a thermostatic autosampler and an AGILENT InfinityLab Poroshell 120 EC-C18 column (4.6 × 150 mm, particle diameter 2.7 µm) with guard column (4.6 × 5 mm, particle diameter 2.7 µm) made up of the same stationary phase and thermostated at 30 °C. The mass spectrometer employed was a Finnigan LXQ linear ion trap (Thermo Scientific, San Jose, CA, USA) equipped with an electrospray ion source (ESI) operating in negative mode.

### Binding assay — K_D_ determination protocol

**Fig. 1:**
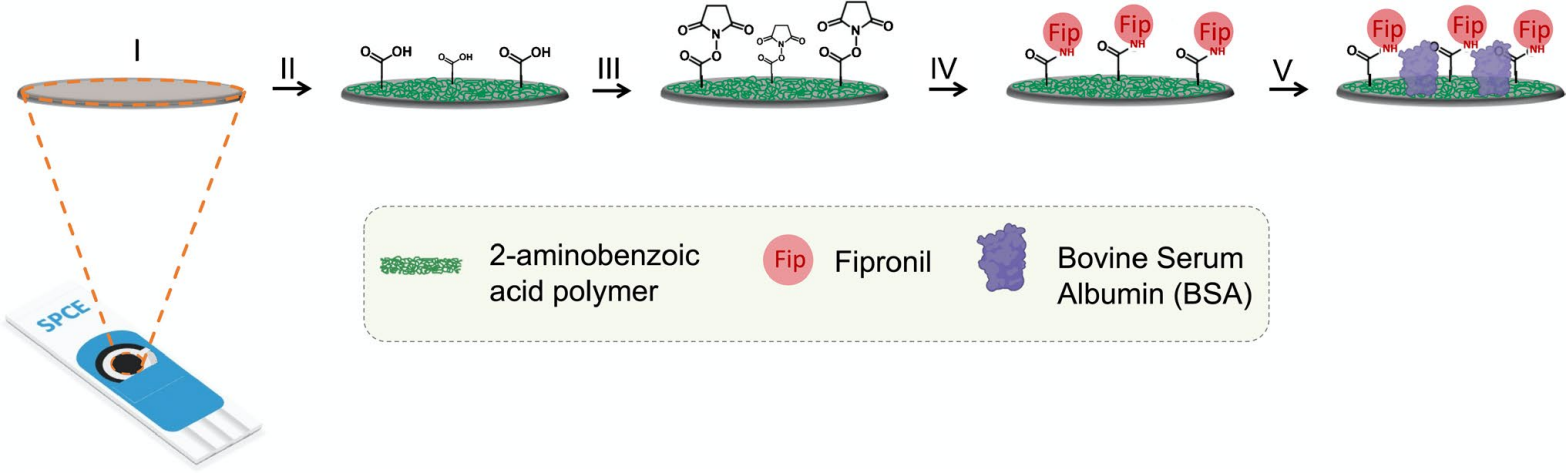
Schematic representation of the different modification steps of the SPCE surface for the determination of the K_D_ of the aptamer-fipronil adduct. I, bare electrode; II, 2-aminobenzoic acid electropolymerization; III, incubation with EDC-NHS reagent; IV, incubation with fipronil; V, incubation with BSA

All SPCEs, before being used, were washed with 1 mL of ethanol and 1 mL of ultrapure water and finally dried with compressed air. Subsequently, 60 µL of the 2-ABA (50 mM) and KCl (0.1 M) solution prepared in H_2_SO_4_ (1 M) were deposited on the SPCE. The polymerization was carried out by applying 10 cycles of cyclic voltammetry starting from an initial potential of 0 V to a final potential of 1 V, at a scan rate equal to 0.05 V/s, cyclic voltammograms of the electropolymerization are shown in Figure S1. This process led to the formation on the working electrode (WE) of a polymer presenting carboxyl groups, in accordance with the proposed mechanism shown in Figure S2. Subsequently, the carboxyl groups were activated by incubating 10 µL of an EDC–NHS reaction mix, in PBS pH 5.6, for 1 h. Afterwards, the electrode surface was incubated overnight with 10 µL of a fipronil solution 40 µM. At the end of this step, fipronil was covalently linked to the electrode surface thank to the formation of amidic bonds. Next, to minimize non-specific binding, the electrode surface was incubated for 15 min with 10 µL of a 1% bovine serum albumin (BSA) solution, prepared in PBS pH 7.4. In order to control the fabrication of the sensor, we performed EIS measurements after each modification step; see Figure S3. Finally, the electrode was ready to carry out the measurements aimed at establishing the apparent dissociation constant (K_D_) of fipronil aptamer. Figure 1 shows a schematic representation of the various steps of SPCE modification.

At this point, 10 µL of the solutions at different concentrations of aptamers FPAP e FPAS prepared in fipronil buffer solution (F-BS: NaCl 100 mM, MgCl_2_ 2 mM, Tris–HCl 20 mM) were incubated for 30 min on modified SPCEs. Then, the SPCEs were incubated for 10 min with the streptavidin-HRP solution (0.75 µg mL^−1^). The electrochemical transduction was performed by adding on the electrode 60 μL of TMB solution, and after 1 min of enzymatic reaction, a chrono-amperometric measurement at 0 V was performed, sampling the reduction current of the oxidized TMB after 55–60 s.

### Electrochemical competitive replacement assay for fipronil detection

**Fig. 2:**
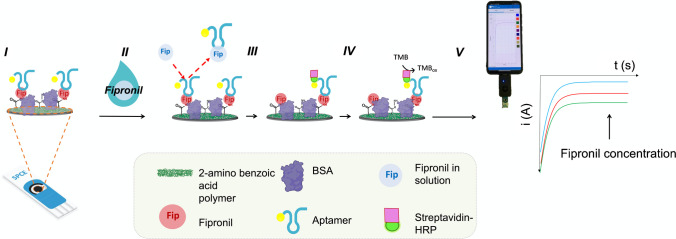
Schematic representation of the competitive replacement assay. I, SPCE modified and ready for the analysis; II, incubation with solution containing fipronil (when the aptamer comes int contact with a sample containing fipronil, it detaches from the electrode surface to bind fipronil in solution); III, incubation with streptavidin-HRP; IV, TMB substrate incubation; V, chrono-amperometric measurement performed with a portable potentiostat managed by a smartphone (increasing fipronil concentration from green to blue)

The procedure for modifying the SPCE for the fipronil determination assay aligns with the modification process described above for measuring K_D_. In this case, to minimize non-specific binding, after optimization, 0.5% BSA solution was used, prepared in PBS at pH 7.4, and incubated on the WE for 5 min. Afterwards, the aptamer was incubated at a concentration of 1 µM. After 30 min of incubation, the electrode surface was rinsed and dried, thus resulting ready for the subsequent assay. The calibration of the sensor response to fipronil was performed by incubating 10 µL of each standard solution at different concentrations for 1 h on the modified SPCEs as previously reported. At the end of this step, the SPCEs were incubated for 10 min with the streptavidin-HRP solution (0.75 µg mL^−1^). The electrochemical transduction was performed by adding on the electrode 60 μL of TMB solution, and after 1 min of enzymatic reaction, a chrono-amperometric measurement at 0 V was performed, sampling the reduction current of the oxidized TMB after 55–60 s. Figure 2 shows a schematic representation of the fipronil assay procedure using the competitive replacement assay, and Figure S4 shows the measurement device including the smartphone and Sensit/SMART potentiostat connected to a SPCE.

### Selectivity and stability evaluation

For the evaluation of the selectivity, the procedure adopted for the analysis was followed by replacing the solutions of fipronil with solutions of known concentration of atrazine, which is a commonly used herbicide and one of the interferent reported in the literature in the quantification of fipronil [42]. Therefore, to determine the selectivity, the assay was conducted with the same modalities adopted for fipronil. Furthermore, the stability of the sensor stored at 4 °C was evaluated. A batch of sensors was prepared and stored ready for the use; the signal at the fixed concentration of 7 μg kg^−1^ was evaluated for 10 days.

### Real samples

In this study, we analyzed two distinct types of honey: one sourced from organic farming and the other one from non-organic farming. The samples were conveniently diluted 1:2 with F-BS prior to analysis and spiked with gradually increasing concentrations of fipronil. To prevent any potential contamination, the sample preparation was conducted in a separate laboratory from the one used for the subsequent analyses. Sample measurements were always accompanied by blank measurements (i.e., matrix without analyte) to evaluate and take into account the matrix effect.

### HPLC–MS analysis

#### Sample preparation procedure

Two-hundred fifty milligrams of each honey sample was purified by solid-phase extraction with an SPE C18 cartridge. For this purpose, the honey samples were diluted in water and loaded onto the SPE cartridge previously conditioned with methanol and water. This was followed by washing with water (10 mL) and elution of fipronil with methanol (5 mL). Finally, after vacuum distillation of the organic solvent with Rotavapor (T < 35 °C) and solubilization of the residue in 1 mL of methanol/water 80/20%, the sample was then subjected to HPLC–MS analysis.

#### HPLC-MS^2^ procedure

The chromatographic separation was carried out on a C18 reverse phase column using a mixture of methanol and water as the mobile phase, at a flow rate of 0.3 mL min^−1^ and with the concentration gradient shown in Table S1. Fipronil was identified in negative mode, isolating the ion having an *m/z* ratio of 435 and corresponding to [M-H]^−^, whose spectrum is shown in Figure S5. This ion was subsequently fragmented obtaining the fragment ions at *m/z* 399 [M-H-HCl]^−^ and at *m/z* 330 [M-H-HCl-CF_3_]^−^ as described in literature [43, 44]. The quantification of fipronil was carried out using the standard additions method and selecting in MS^2^ the most intense fragment ion at *m/z* 399 (Figure S6a). By way of example, Figure S6b shows the chromatograms and the mass spectrum obtained in the HPLC-MS^2^ analysis of a solution at known fipronil content.

## Results and discussion

### K_D_ determination

Binding assays were performed to establish the dissociation constants of the fipronil aptamer adducts.

The sequence described in the literature, which comprises the primers used during the selection procedure, has been taken into consideration. Simultaneously, the same sequence was evaluated without primers, considering that the variable portion, i.e., the portion without primer, is typically involved in binding with the target.

**Fig. 3:**
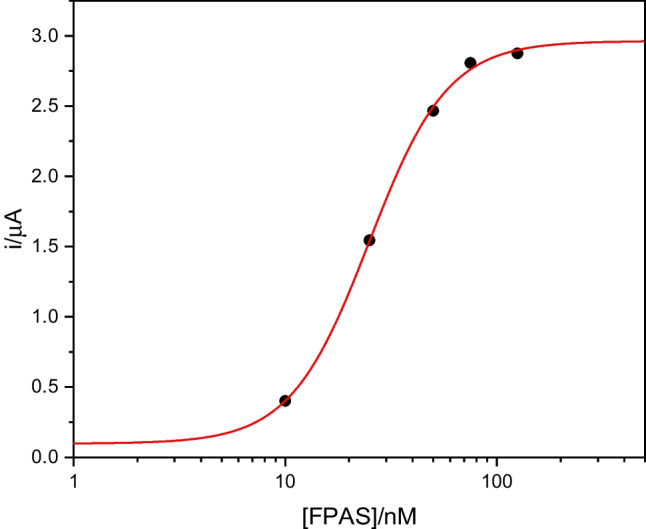
Binding curve of FPAS vs. fipronil (10, 25, 50, 75, 125 nM)

In Table 2 are reported the K_D_ values, while Fig. 3 and Figure S7 show the related titration curves. The best fittings, characterized by coefficients of determination (*R*^2^) = 0.99, were obtained with the logistic model.

**Table 2:** Dissociation constants (*K*_D_) of the fipronil aptamer adducts

Aptamer	K_D_ (nM)
FPAP	94.3 ± 0.5
FPAS	24.9 ± 1.7

The K_D_ data show that the affinity of FPAS towards fipronil is higher than that calculated for FPAP. These results confirm the advantage of utilizing the aptamer without the primer regions in terms of affinity. Consequently, based on the K_D_ values, FPAS was selected for the subsequent experimental activities.

### Fipronil quantification by a competitive replacement assay

Encouraged by the results of the binding assay of FPAS, an electrochemical aptasensor for fipronil quantification using a competitive replacement assay was developed. The principle of the assay is illustrated in Fig. 2. This type of approach has already been successfully employed in literature for the quantification of small molecules such as neomycin or aflatoxin B [40, 41]. Here, we exploited the competitive replacement of the aptamer bound to the target immobilized on the surface with the free fipronil molecules in solution. Aptamers exhibit a higher preference for binding targets in solution rather than immobilized on a surface, because of accessibility, conformational flexibility, and more favorable electrostatic interactions [45]. Hence, this preference was exploited in the design of this assay, and when the aptamer comes into contact with a sample containing fipronil, it detaches from the electrode surface to bind fipronil in solution. Subsequently, through a washing step, the aptamer is eliminated from the electrode surface, causing a decrease in the reduction current of the oxidized TMB as the concentration of fipronil in solution increases.

**Fig. 4:**
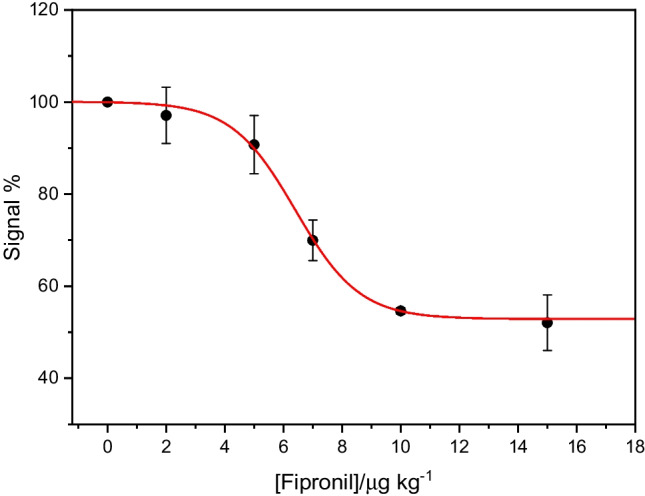
Calibration curve of the signal obtained from chrono-amperometric measurements with the aptasensor on standard solutions with increasing fipronil concentration. The error bar is the standard deviation from a minimum of three measurements for each concentration

Figure 4 shows the calibration curve obtained with increasing concentration of fipronil; the percentage signal decreases as the fipronil concentration increases. The model that best fits the data obtained is a dose–response model represented, with an *R*^2^ of 0.999, by Eq. 1.

1 $$y=52.90+ \frac{100.09-52.90}{1+ {10 }^{(6.41-x)(-0.40)}}$$

**Fig. 5:**
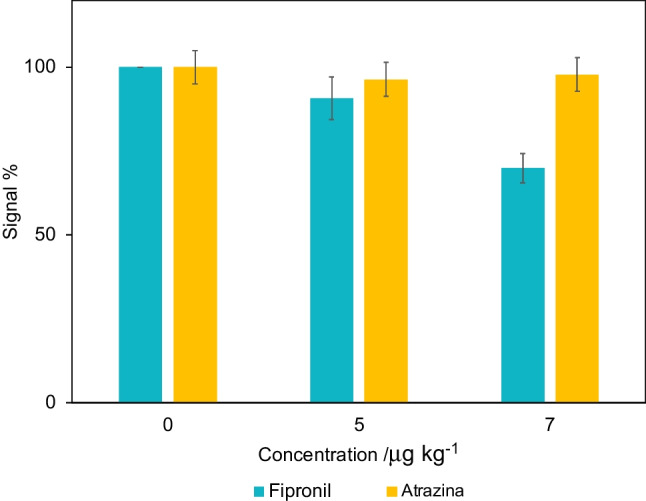
Decrease of the signal obtained from chrono-amperometric measurements with the aptasensor on standard solutions with increasing concentrations of fipronil and atrazine

The limit of detection (LOD), calculated as three times the standard deviation of the blank signal divided by the slope calculated for the linear range, was 1.07 μg kg^−1^, while the limit of quantification (LOQ) was 3.21 μg kg^−1^. This confirms the high sensitivity of the response of the developed aptasensor, suitable for detecting quantities of fipronil even below the maximum residual level established by the European Union in the main food products, which is 5 μg kg^−1^. The selectivity of the aptasensor with respect to an herbicide product such as atrazine was evaluated. Atrazine was selected due to its resemblance to fipronil, characterized by its small molecular size and stereochemical arrangement, making it one of the most frequently employed compounds to test the selectivity of the developed sensor. The results obtained by comparing the response of the sensor in solutions of known concentration of fipronil and atrazine are summarized in Fig. 5. The aptasensor stability was tested measuring its activity during 10 days of refrigerated storage. We recorded a maximum of 10% of sensor response decrease, indicating that the sensor can be used for up to 10 days after fabrication without major loss of efficiency.

Regarding the advances in the field of fipronil analysis, Table 3 compares the aptasensors for the detection of fipronil proposed by the literature in terms of analytical performance. It can be seen that the smartphone aptasensor here reported successfully competes with the other present in literature; moreover, it advances a portable analysis of fipronil, compared to the other approaches which still employ classic instrumentation with the correlated limitations.

**Table 3:** Comparison of the analytical performance of the developed aptasensor with that of other aptasensors for the determination of fipronil

Transduction method	Range	LOD	Sample	Reference
Fluorescence	5–500 nM	105 nM	Water	[42]
Fluorescence	25–300 μg kg^−1^	53.8 μg kg^−1^ (123 nM)	Eggs	[46]
Fluorescence	10–100 nM	3 nM	Corn, honey, water	[47]
Electrochemistry	0.1 ng/mL–10 µg/mL	0.17 nM	Water	[48]
Fluorescence	(5–70) nM	3.4 nM	Cabbage, cucumber	[49]
Electrochemistry (smartphone-based)	2–10 μg kg^−1^	1.07 μg kg^−1^ (2.45 nM)	Honey	This work

## Application of the competitive assay to food samples

To verify whether the developed biosensor could be employed in the analysis of real food samples, we proceeded to the analysis of fipronil in samples of honey.

First, the two honey samples were subjected to HPLC–MS analysis to determine the initial fipronil content. The honey sample sourced from organic farming did not show any detectable presence of fipronil, or if present, it was below the limit of detection (0.19 μg kg^−1^) of the HPLC–MS method. In contrast, the non-organic farming honey exhibited a fipronil content of 2.35 ± 0.35 μg kg^−1^. These data were used as starting point to evaluate the results provided by the biosensor.

The honey samples underwent parallel analysis using a biosensor, employing the standard addition method. Fipronil was incrementally added to the samples, ensuring concentrations that fell within the dynamic response range of the sensor and were comparable to the maximum residue level (MRL) mandated by European legislation for honey.

The summarized results for the organic farming honey and non-organic farming honey are presented in Table 4.

**Table 4:** Comparison between the theoretical concentration of fipronil and that determined experimentally in real samples of honey, analyzed as they are and spiked with known quantities of fipronil

Fipronil (μg kg^−1^)	Expected fipronil concentration (μg kg^−1^)	Biosensor quantification (μg kg^−1^)
Organic farming honey		
0.00	< LOD	/
5.00	5.00	5.84 ± 0.23
7.00	7.00	7.64 ± 0.82
Non-organic farming honey		
0.00	2.35 ± 0.35	3.24 ± 0.90
5.00	7.35 ± 0.35	7.11 ± 0.34
7.00	9.35 ± 0.35	9.11 ± 1.25

These results confirm the reliability of the biosensor as it consistently produces results that align with those obtained through the HPLC–MS method. Moreover, the accuracy of the biosensor’s measurements is demonstrated even in spiked samples, meeting the expected concentrations.

## Conclusions

This study demonstrates that a competitive replacement assay, based on aptamers, is a suitable approach for quantifying fipronil in food matrices. This assay format addresses the challenges associated with detecting small molecules. By utilizing a simple and cost-effective electrochemical platform that can be used with a smartphone, the proposed assay becomes readily accessible, making it a user-friendly and portable detection system for detecting fipronil.

With a limit of detection (LOD) of 1.07 μg kg^−1^, the assay proves to be highly sensitive. To confirm its applicability in real food samples, honey samples were tested. The successful results provide confidence in the reliability of the biosensor for fipronil detection in various food matrices. This indicates that this aptasensor could be a valuable tool for screening and monitoring fipronil contamination in food products, contributing to food safety efforts. Further research and validation on a wider range of food samples would help strengthen its applicability and broaden its potential impact in food safety monitoring.

### Supplementary Information

Below is the link to the electronic supplementary material.

Supplementary file1 (DOCX 811 KB)

## References

[1] Sabzevari S, Hofman J (2022). A worldwide review of currently used pesticides’ monitoring in agricultural soils. Sci Total Environ.

[2] Damalas CA, Eleftherohorinos IG (2011). Pesticide exposure, safety issues, and risk assessment indicators. Int J Environ Res Public Health.

[3] Tudi M, Daniel Ruan H, Wang L, Lyu J, Sadler R, Connell D, et al. Agriculture development, pesticide application and its impact on the environment. Int J Environ Res Public Health 2021;18. 10.3390/ijerph18031112. 10.3390/ijerph18031112 PMC7908628 33513796

[4] Fang W, Peng Y, Muir D, Lin J, Zhang X (2019). A critical review of synthetic chemicals in surface waters of the US, the EU and China. Environ Int.

[5] Czaja K, Struciński P, Korcz W, Minorczyk M, Hernik A, Wiadrowska B (2020). Alternative toxicological methods for establishing residue definitions applied for dietary risk assessment of pesticides in the European Union. Food Chem Toxicol.

[6] Ioannidou S, Cascio C, Gilsenan MB (2021). European Food Safety Authority open access tools to estimate dietary exposure to food chemicals. Environ Int.

[7] Scientific criteria to ensure safe food (2003). Washington.

[8] Frelih-Larsen A, Chivers C-A, Herb I, Mills J, Reed M (2023). The role of public consultations in decision-making on future agricultural pesticide use: insights from European Union’s farm to fork strategy public consultation. J Environ Planning Policy Manage.

[9] McGinley J, Healy MG, Ryan PC, Harmon O’Driscoll J, Mellander P-E, Morrison L (2023). Impact of historical legacy pesticides on achieving legislative goals in Europe. Sci Total Environ.

[10] Thorat T, Patle BK, Wakchaure M, Parihar L (2023). Advancements in techniques used for identification of pesticide residue on crops. J Nat Pestic Res.

[11] Schleiffer M, Speiser B (2022). Presence of pesticides in the environment, transition into organic food, and implications for quality assurance along the European organic food chain – a review. Environ Pollut.

[12] Reich H, Triacchini GA, European Food Safety Authority (EFSA) (2018). Occurrence of residues of fipronil and other acaricides in chicken eggs and poultry muscle/fat. EFSA J.

[13] Anagnostopoulos C, Ampadogiannis G, Bempelou E, Liapis K, Kastellanou E (2020). The 2017 fipronil egg contamination incident: the case of Greece. J Food Saf.

[14] Wang X, Martínez MA, Wu Q, Ares I, Martínez-Larrañaga MR, Anadón A (2016). Fipronil insecticide toxicology: oxidative stress and metabolism. Crit Rev Toxicol.

[15] Tingle CCD, Rother JA, Dewhurst CF, Lauer S, King WJ (2003). Fipronil: environmental fate, ecotoxicology, and human health concerns. Rev Environ Contam Toxicol.

[16] Zhou Z, Wu X, Lin Z, Pang S, Mishra S, Chen S (2021). Biodegradation of fipronil: current state of mechanisms of biodegradation and future perspectives. Appl Microbiol Biotechnol.

[17] Farder-Gomes CF, Fernandes KM, Bernardes RC, Bastos DSS, Martins GF, Serrão JE (2021). Acute exposure to fipronil induces oxidative stress, apoptosis and impairs epithelial homeostasis in the midgut of the stingless bee Partamona helleri Friese (Hymenoptera: Apidae). Sci Total Environ.

[18] Mendonça JDS, De Almeida JCN, Vieira LG, Hirano LQL, Santos ALQ, Andrade DV (2023). Mutagenicity, hepatotoxicity, and neurotoxicity of glyphosate and fipronil commercial formulations in Amazon turtles neonates (Podocnemis expansa). Sci Total Environ.

[19] Li X, Ma W, Li H, Zhang Q, Ma Z (2020). Determination of residual fipronil and its metabolites in food samples: a review. Trends Food Sci Technol.

[20] Reasoned opinion on the review of the existing maximum residue levels (MRLs) for fipronil according to Article 12 of Regulation (EC) No 396/2005. EFSA J n.d. 10.2903/j.efsa.2012.2688. 10.2903/j.efsa.2012.2688 PMC13136677 42088879

[21] Kaur R, Mandal K, Kumar R, Singh B (2015). Analytical method for determination of fipronil and its metabolites in vegetables using the QuEChERS method and gas chromatography/mass spectrometry. J AOAC Int.

[22] Hafeez A, Tawab IA, Iqbal S (2016). Development and validation of an HPLC method for the simultaneous determination of fipronil, chlorfenapyr, and pyriproxyfen in insecticide formulations. J AOAC Int.

[23] Charalampous AC, Liapis KS, Bempelou ED (2019). Fipronil in eggs. Is LC-MS/MS the only option? A comparison study of LC-MS/MS and GC-ECD for the analysis of fipronil. J Chromatogr B.

[24] del Valle M (2021). Sensors as green tools in analytical chemistry. Curr Opin Green Sustain Chem.

[25] Tu Q, Hickey ME, Yang T, Gao S, Zhang Q, Qu Y (2019). A simple and rapid method for detecting the pesticide fipronil on egg shells and in liquid eggs by Raman microscopy. Food Control.

[26] Zhou XH (2020). Determination of fipronil and its metabolites in eggs by indirect competitive ELISA and lateral-flow immunochromatographic strip. Biomed Environ Sci..

[27] Pohanka M (2022). Aptamers in Electrochemical Biosensors. Int J Electrochem Sci.

[28] Radom F, Jurek PM, Mazurek MP, Otlewski J, Jelen F (2013). Aptamers: molecules of great potential. Biotechnol Adv.

[29] Althomali RH, Abdu Musad Saleh E, Gupta J, Mohammed Baqir Al-Dhalimy A, Hjazi A, Hussien BM (2023). State-of-the-art of portable (bio)sensors based on smartphone, lateral flow and microfluidics systems in protozoan parasites monitoring: a review. Microchem J.

[30] Costa-Rama E, Fernández-Abedul MT (2021). Paper-based screen-printed electrodes: a new generation of low-cost electroanalytical platforms. Biosensors.

[31] Toniolo R, Dossi N, Giannilivigni E, Fattori A, Svigelj R, Bontempelli G (2020). Modified screen printed electrode suitable for electrochemical measurements in gas phase. Anal Chem.

[32] Li G, Wang X, Row KH (2018). Magnetic molecularly imprinted polymers based on silica modified by deep eutectic solvents for the rapid simultaneous magnetic-based solid-phase extraction of *Salvia*
*miltiorrhiza*
*bunge*, *Glycine*
*max* (*Linn.*) *Merr* and *green*
*tea*. Electrophoresis.

[33] Prante M, Segal E, Scheper T, Bahnemann J, Walter J. Aptasensors for point-of-care detection of small molecules. Biosensors 2020;10. 10.3390/bios10090108. 10.3390/bios10090108 PMC7559136 32859075

[34] Pfeiffer F, Mayer G. Selection and biosensor application of aptamers for small molecules. Front Chem 2016;4. 10.3389/fchem.2016.00025. 10.3389/fchem.2016.00025 PMC4908669 27379229

[35] Svigelj R, Dossi N, Grazioli C, Toniolo R (2022). Paper-based aptamer-antibody biosensor for gluten detection in a deep eutectic solvent (DES). Anal Bioanal Chem.

[36] Wang X, Cohen L, Wang J, Walt DR (2018). Competitive immunoassays for the detection of small molecules using single molecule arrays. J Am Chem Soc.

[37] Svigelj R, Zuliani I, Grazioli C, Dossi N, Toniolo R (2022). An effective label-free electrochemical aptasensor based on gold nanoparticles for gluten detection. Nanomaterials.

[38] Cheng S, Shi F, Jiang X, Wang L, Chen W, Zhu C (2012). Sensitive detection of small molecules by competitive immunomagnetic-proximity ligation assay. Anal Chem.

[39] Du P, Jin M, Chen G, Zhang C, Jiang Z, Zhang Y (2016). A competitive bio-barcode amplification immunoassay for small molecules based on nanoparticles. Sci Rep.

[40] de-los-Santos-Álvarez N, Lobo-Castañón MJ, Miranda-Ordieres AJ, Tuñón-Blanco P (2007). Modified-RNA aptamer-based sensor for competitive impedimetric assay of neomycin B. J Am Chem Soc.

[41] Wang C, Zhao Q (2019). A competitive thrombin-linked aptamer assay for small molecule: aflatoxin B(1). Anal Bioanal Chem.

[42] Hong K, Sooter L (2017). In vitro selection of a single-stranded DNA molecular recognition element against the pesticide fipronil and sensitive detection in river water. IJMS.

[43] Raju KSR, Taneja I, Rashid M, Sonkar AK, Wahajuddin M, Singh SP (2016). DBS-platform for biomonitoring and toxicokinetics of toxicants: proof of concept using LC-MS/MS analysis of fipronil and its metabolites in blood. Sci Rep.

[44] Qu L, Qi X, Zhao L, Zhang Y, Zhuge R, Hao Z (2023). Development, validation, and use of a monitoring method for fipronil and its metabolites in chicken eggs by QuEChERS with online-SPE-LC-Q/Orbitrap analysis. Rapid Comm Mass Spectrometry.

[45] Daniel C, Roupioz Y, Gasparutto D, Livache T, Buhot A (2013). Solution-phase vs surface-phase aptamer-protein affinity from a label-free kinetic biosensor. PLoS ONE.

[46] Kim T-Y, Lim JW, Lim M-C, Song N-E, Woo M-A (2020). Aptamer-based fluorescent assay for simple and sensitive detection of fipronil in liquid eggs. Biotechnol Bioprocess Eng.

[47] Zhang J, Feng T, Zhang J, Liang N, Zhao L (2021). Fluorescence assay for the sensitive detection of fipronil based on an “on–off” oxidized SWCNH/aptamer sensor. Anal Methods.

[48] Huang H, Zhang C, Zhou J, Wei D, Ma T, Guo W, et al. Label-free aptasensor for detection of fipronil based on black phosphorus nanosheets. Biosensors 2022;12. 10.3390/bios12100775. 10.3390/bios12100775 PMC9599224 36290913

[49] Trinh KH, Kadam US, Rampogu S, Cho Y, Yang K-A, Kang CH (2022). Development of novel fluorescence-based and label-free noncanonical G4-quadruplex-like DNA biosensor for facile, specific, and ultrasensitive detection of fipronil. J Hazard Mater.

