# Supplementary material for: A smartphone aptasensor for fipronil detection in honey samples
Source: Anal Bioanal Chem. 2023 Nov 9;416(2):397–405. doi: 10.1007/s00216-023-05026-6 (PMC10761377; doi:10.1007/s00216-023-05026-6)
Supplement: Supplementary file 1 — Supplementary file1 (DOCX 811 KB) [file 216_2023_5026_MOESM1_ESM.docx]

**Supporting information**

**A Smartphone Aptasensor for Fipronil Detection in Honey Samples**

Rossella Svigelj^1^*, Noemi Dassi^1^, Andrea Gorassini^2^, Rosanna Toniolo^1^*

^1^Department of Agrifood, Environmental and Animal Science, University of Udine, Udine, Italy;

^2^Department of Humanities and Cultural Heritage, University of Udine, Udine, Italy;

*corresponding authors:

[rossella.svigelj@uniud.it](mailto:rossella.svigelj@uniud.it)

[rosanna.toniolo@uniud.it](mailto:rosanna.toniolo@uniud.it)

**Figure S1.** Electropolymerization of 2-aminobenzoic acid on the electrode surface.


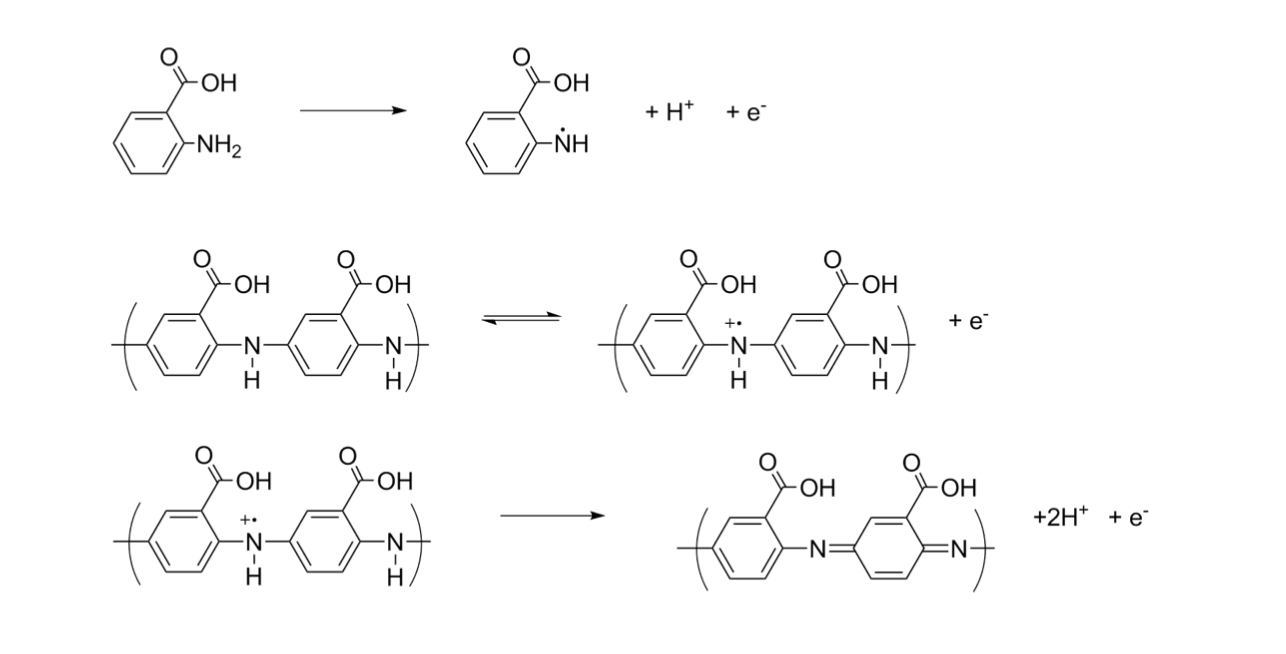


**Figure S2.** Polymerization process of 2-aminobenzoic acid: formation of the radical starting from the monomer; formation of the dimeric radical cation, and formation of the iminoquinone dimer.

**Figure S3.** Nyquist plots recorded in a PBS solution containing 3 mM KCl, 2 mM [Fe(CN)_6_]^3−^ and 2 mM [Fe(CN)_6_]^4−^ at SPCE after each of the modification steps involved in the preparation of the aptasensor . The electropolymerization of 2-ABA (orange) lead to an increase in the charge transfer resistance, R_ct_ compared to the bare SPCE (black), this behavior is in line with the generation of the polymer. The introduction of fipronil on the surface (red) facilitates the charge transfer. The blocking step with BSA (yellow) lead to an increase in the charge transfer resistance, lastly, we observed a further increase in R_ct_ upon the binding of the aptamer (green) as a consequence of the electrostatic repulsion by the polyanionic phosphate backbone of the DNA.

**Table S1.** Concentration gradient of the mobile phase during HPLC separation.

| Time (min) | MeOH (%) | H_2_O (%) |
| --- | --- | --- |
| 0-12 | 80 | 20 |
| 15-21 | 100 | 0 |
| 23-30 | 80 | 20 |

**Figure S4. Measurement device including smartphone and Sensit/SMART potentiostat connected to a SPCE.**


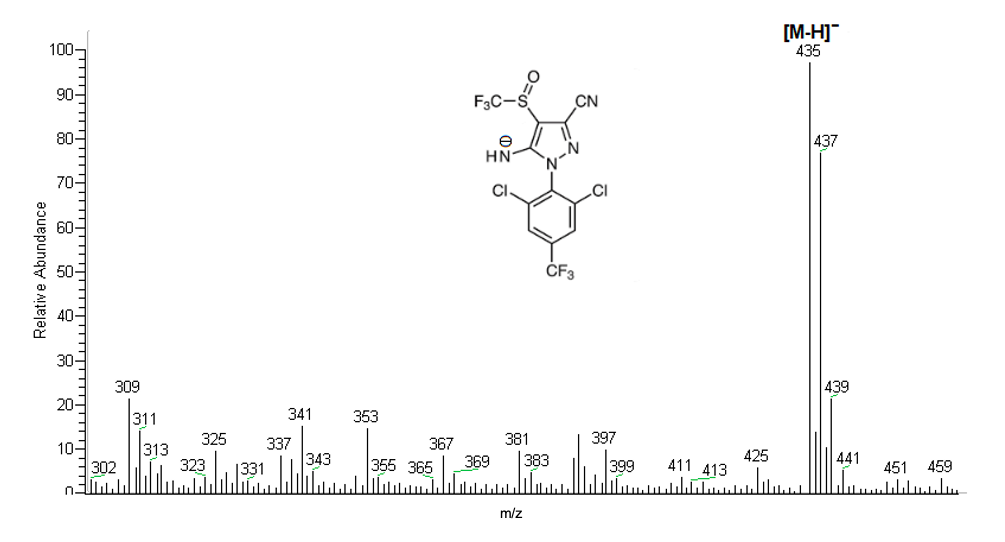


**Figure S5.** Fipronil mass spectrum.

**Figure S6.** a) mass spectrum resulting from the fragmentation of the quasi-molecular ion of fipronil.

b) chromatogram resulting from TIC acquisition and chromatogram resulting from the selection of the ion at *m/z* 399.

**Figure S7.** Binding curve of FPAP vs. fipronil (10, 25, 75, 100, 125 nM).
